# Integrated Analysis of *Pithecellobium dulce* Leaf Fractions for Ex Vivo Antioxidant, DNA Damage Protective, and Afzelin‐Mediated Anti‐Inflammatory Potentials via In Vivo and In Silico Approaches

**DOI:** 10.1155/omcl/5529212

**Published:** 2026-10-19

**Authors:** Masuma Akter, Mst. Shahnaj Parvin, Md. Ekramul Islam

**Affiliations:** ^1^ Department of Pharmacy Faculty of Science University of Rajshahi Rajshahi 6205 Bangladesh ru.ac.bd; ^2^ School of Natural Sciences Faculty of Science and Engineering Macquarie University Sydney 2109 New South Wales Australia mq.edu.au

**Keywords:** afzelin, antioxidants, DNA damage, molecular docking, oxidative stress

## Abstract

As medicinal plants offer promising alternatives to synthetic drugs, this study explored the ex vivo antioxidant and in vitro anti‐inflammatory capacities of *Pithecellobium dulce* leaf extracts, alongside the in vivo anti‐inflammatory potential of its bioactive flavonoid, afzelin, using experimental and computational approaches. In erythrocytes, 2,2^′^‐azobis(2‐amidinopropane)dihydrochloride (AAPH) induced a time‐dependent increase in hemolysis, malondialdehyde (MDA) formation, and depletion of cytosolic glutathione (GSH) and superoxide dismutase (SOD). The chloroform fraction (CHF) and ethyl acetate fraction (EAF) mitigated hemolysis and lipid peroxidation, enhanced GSH and SOD levels, lowered MDA levels compared with the AAPH group, and also protected P^UC19^ plasmid DNA from oxidative damage. In anti‐inflammatory assays, EAF and CHF inhibited heat‐induced red blood cell (RBC) lysis by 57.31% ± 0.80% and 51.39% ± 0.27% and egg albumin denaturation by 39.74% ± 0.77% and 37.20% ± 2.16%, respectively, compared with 61.52% ± 0.58% for acetylsalicylic acid. Afzelin (50 mg/kg body weight) reduced carrageenan‐induced paw edema by 56.16%, versus 70.83% for diclofenac sodium. Bioinformatics analysis identified 38 potential anti‐inflammatory protein targets of afzelin, of which 11 were further examined via molecular docking. Docking results revealed favorable binding energies for all targets, with the highest affinity observed for JAK3 (−9.60 kcal/mol) compared to native ligands. These computational results suggest that afzelin can form stable, energetically favorable interactions with multiple key proinflammatory proteins. Overall, these findings indicate that *P. dulce* leaf extracts possess significant antioxidant and anti‐inflammatory activities, and afzelin showed predicted interactions with multiple kinase and nonkinase inflammation‐associated proteins.

## 1. Introduction

Oxidative stress and chronic inflammation are basic pathological processes associated with a variety of human diseases, including cancer, cardiovascular diseases, metabolic diseases, and neurodegenerative diseases [1–3]. Increased reactive oxygen species (ROS) generation not only leads to alteration of redox homeostasis but it also damages macromolecules, including lipids, proteins, and DNA, and eventually leads to inflammatory reactions and apoptosis [4–6]. Therefore, discovering natural bioactive products that can act as antioxidants and anti‐inflammatory agents is a significant treatment strategy.

Medicinal plants are a rich source of phytochemicals that modulate pathways of oxidative stress and inflammation. *Pithecellobium dulce*, more commonly known as Jilapi babul, has historically been utilized in folk medicine for the characteristics of anti‐inflammatory, antimicrobial, hepatoprotective, and wound‐healing characteristics [7–12]. In particular, its leaves have been targeted for their phytoconstituent properties, including flavonoids, tannins, and phenolic glycosides, which potentially provide their protective effect against oxidative stress and inflammation [13–16]. Afzelin (kaempferol‐3‐O‐rhamnoside) has received attention due to its reported antioxidant, anticancer, and immunomodulatory properties [17–19]. However, its specific role in the regulation of inflammation‐related signaling pathways has not yet been fully established.

Integrative methods, including ex vivo antioxidant assays, DNA damage protection studies, in vivo studies, and in silico docking studies, provide a complete framework to evaluate the pharmacological potential of plant‐derived fractions and their bioactive components. These multifaceted studies are beneficial in compartmentalizing traditional claims and demonstrating molecular mechanisms of action of exposure, and the goal is to further develop phytoconstituents as therapeutic scaffolds.

Previously, we have reported the in vitro antioxidant and antibacterial activity of *P. dulce* leaves [20] as well as the isolation of the compound afzelin from the ethyl acetate fraction (EAF) of this plant, and characterization was confirmed via thin‐layer chromatography (TLC) and nuclear magnetic resonance (NMR) [21], demonstrating its antioxidant, DNA damage protective, and antiproliferative activity. The present study focuses on the investigation of the EAF and chloroform fraction (CHF) of *P. dulce* leaves against 2,2^′^‐azobis(2‐amidinopropane)dihydrochloride (AAPH)‐induced oxidative and afzelin‐mediated anti‐inflammatory potentials via in vivo and in silico approaches.

This study was therefore designed to comprehensively investigate the antioxidant, DNA damage‐protective, and anti‐inflammatory potentials of *P. dulce* leaf fractions (EAF and CHF), with a special emphasis on afzelin, a bioactive flavonoid previously isolated from the EAF, through combined in vivo and in silico approaches. Ex vivo assays were employed to assess antioxidant capacity and DNA protective effects, while *in vivo* experiments evaluated the anti‐inflammatory efficacy of afzelin. Furthermore, molecular docking was performed to delineate the binding interactions of afzelin with key proinflammatory targets. Unlike previous studies that have focused primarily on crude extracts or limited bioactivity assays, this work integrates biochemical, pharmacological, and computational analyses to provide mechanistic insights into afzelin‐mediated anti‐inflammatory pathways. The novelty of this research lies in integrating experimental evaluation of afzelin‐mediated anti‐inflammatory activity with computational analysis of its predicted interactions with inflammation‐associated targets, thereby positioning *P. dulce* as a promising source of lead compounds for the management of inflammation‐driven disorders.

## 2. Materials and Methods

### 2.1. Chemicals and Reagents

TBA, 5,5^′^‐dithiobis (2‐nitrobenzoic acid) (DTNB), and EDTA were supplied from Sigma–Aldrich. The source of the pUC19 plasmid DNA was Thermo Fisher Scientific. Other reagents, such as diclofenac sodium, ascorbic acid, carrageenan, and ethidium bromide, were also used. AAPH was obtained from FUZIFILM Wako Pure Chemical Corporation. Additional analytical‐grade substances used in the assays were acquired from Sigma–Aldrich and Roche.

### 2.2. Collection, Extraction, and Fractionation of Plant Materials

The plant parts were collected from the local area of Kumarkhali, Kushtia, and authenticated by a taxonomist from the University of Rajshahi′s Botany Department. A voucher specimen was deposited in the National Herbarium, Dhaka, Bangladesh, under Accession Number 48085. The healthy leaves were then cleaned, dried, and ground into a coarse powder using a mechanical grinder. Approximately 1.5 kg of this leaf powder was then subjected to n‐hexane extraction to remove non‐polar pigments and chlorophyll. This fraction was discarded because its high chlorophyll content could interfere with spectrophotometric assays and potentially complicate subsequent chromatographic separation.

The remaining residue was soaked in 3.5 L of methanol with continuous shaking and stirring for 12 days. The mixture was subsequently filtered through cotton and Whatman No. 1 filter paper. The crude methanolic extract (CME) was collected using a rotary evaporator. This extract was then fractionated using n‐hexane (effectively removing chlorophylls, low‐polar compounds, and fatty substances with n‐hexane before disposal), chloroform (CHF), and ethyl acetate (EAF).

### 2.3. Ex Vivo Antioxidant Role Against Oxidative Damage

AAPH is an azo compound that generates aqueous peroxyl radicals through thermal decomposition, leading to free radical chain oxidation that is mainly responsible for the development of chronic diseases [22, 23]. To evaluate the extracts’ (specifically EAF and CHF) ability to normalize or scavenge the oxidative damage on human erythrocytes, several techniques were employed. These included assessing the protection of erythrocytes and DNA, inhibiting lipid peroxidation, measuring glutathione (GSH) levels, and determining superoxide dismutase (SOD) activity.

#### 2.3.1. Erythrocyte Suspension Preparation

A healthy participant, who was briefed on the study′s purpose and abstained from any drug for 7 days before the venipuncture, provided blood samples (5–10 mL each). To separate the erythrocytes, the citrated blood samples were centrifuged for 15 min at 4000 rpm. To reach the required hematocrit level (5%), the samples were resuspended in the same buffer after being cleaned three times with phosphate‐buffered saline (PBS).

#### 2.3.2. Protective Effect of EAF and CHF on Human Erythrocyte Induced by AAPH

The protective effects of EAF and CHF on erythrocytes against AAPH‐induced oxidative damage were evaluated using a modified method from Yang et al. [24, 25]. First, preincubation of a 5% hematocrit erythrocyte solution was performed at 37°C with PBS (control) and 100 μg/mL of ascorbic acid, along with varying concentrations of EAF (25–100 μg/mL) and CHF (50–200 μg/mL) from *P. dulce* leaves. For time‐dependent analysis, the erythrocyte samples were then incubated for 6 h with AAPH (50 mM). A reference value was also included using an equal volume of erythrocytes in hypotonic buffer to achieve 100% hemolysis under the same conditions. Samples were collected at regular intervals, diluted with two milliliters of PBS, and centrifuged at 4000 rpm for 10 min. After collecting the supernatant, the absorbance at 540 nm was measured. The experiment for all samples was performed in triplicate, and results were expressed as the mean ± standard deviation (SD). Each assay included appropriate positive and negative controls: ascorbic acid served as the reference anti‐inflammatory drug, and a reagent blank was run simultaneously, and absorbance readings were corrected against blank values to eliminate background interference and ensure data accuracy. The ratio of the sample′s absorbance at 540 nm to that of the reference was used to assess the degree of hemolysis.

% of hemolysis=absorbance of sampleabsorbance of reference×100.

#### 2.3.3. Quantitative Estimation of Malondialdehyde (MDA) Level on Erythrocyte

MDA, a byproduct of lipid peroxidation, was quantified using the TBARS assay, as described by Yang et al. [24, 25]. Red blood cell (RBC) suspensions (5% hematocrit) were preincubated with varying concentrations of the test samples (50, 100, and 200 μg/mL), followed by exposure to 50 mM AAPH in PBS for 6 h. The reaction mixture (1 mL) was treated with 300 μL of 0.44 M H_3_PO_4_ and 750 μL of 0.67% TBA and then incubated at 95°C for 1 h. After cooling in an ice bath, 450 μL of 20% trichloroacetic acid was added. Samples were centrifuged at 4000 rpm for 10 min, and the absorbance was measured at 532 nm. All experiments were performed in triplicate, and results were expressed as the mean ± SD. Each assay included an appropriate positive and negative control. Ascorbic acid was used as the antioxidant standard, and corresponding reagent blanks were run simultaneously, and absorbance readings were corrected against blank values to eliminate background interference and ensure data accuracy. Following Beer–Lambert Law, the MDA level was determined using a molar extinction coefficient of 156 mM^−1^ cm^−1^ and reported as pmol/g Hb. Here, the hemoglobin (Hb) concentration was used as a normalization parameter for MDA quantification to account for variations in erythrocyte content across samples. This approach ensures that lipid peroxidation levels are expressed relative to the cellular mass, thereby improving the accuracy and comparability of oxidative stress measurements.

MDA level (pmol/g Hb)=absorbance of sample156 mM−1 cm−1×dilution factor.

#### 2.3.4. Estimation of GSH Content in Erythrocytes

The reduced GSH content in RBCs was calculated using DTNB in accordance with a standard protocol [24–26]. RBC suspensions were exposed to AAPH for 6 h in the presence of *P. dulce* extracts at concentrations of 100 and 200 µg/mL, with ascorbic acid used as the positive control. To precipitate the cell lysate, 0.6 mL of metaphosphoric acid solution (composed of 1.67 g of HPO_3_ [metaphosphoric acid], 0.2 g of EDTA, and 30 g of sodium chloride dissolved in 100 mL of water) was added. The precipitated protein was then separated by centrifugation at 5000 rpm for 10 min. Next, 0.45 mL of the supernatant was mixed with 0.45 mL of 300 mM Na_2_HPO_4_ (pH 8.0). After that, both in the sample and the blank, 100 μL of DTNB solution (20 mg of DTNB in 100 mL of 1% citrate solution) was added, and the absorbance was recorded at 412 nm. The GSH levels were reported in μmol/g Hb. All measurements were performed in triplicate and repeated in at least three independent experiments.

#### 2.3.5. Evaluation of Erythrocyte SOD Activity

SOD activity was assessed using an improved method by Marklund and Marklund [27]. In this approach, superoxide (O_2_^−^•) is generated indirectly at alkaline pH through the auto‐oxidation of pyrogallol. SOD interacts with superoxide, which reduces the rate of formation of o‐hydroxybenzoquinone and other polymer products [28]. The SOD activity of EAF and CHF was evaluated by preincubating them at 100 and 200 µg/mL concentrations with RBC suspension at 37°C for 30 min, followed by the addition of 50 mM AAPH. After that, the reaction mixture was kept at 37°C for 6 h in order to facilitate time‐dependent hemolysis. Samples were taken out and centrifuged every 2 h. Then, the reaction mixture consisted of 2.5 mL of solution A (containing 0.1 mol/L tris‐HCl buffer solution with 1 mmol/L EDTA at pH 8.20), 2.0 mL of distilled water, and 50 μL of the test sample, to which 50 μL of pyrogallol solution B (4.5 mol/L pyrogallol solution in 10 mmol/L HCl) was added. Immediately after mixing, the decrease in the rate of pyrogallol auto‐oxidation was recorded at 325 nm. Enzyme activity was expressed as units per milligram of Hb (U/mg Hb), with Hb content of the erythrocyte lysate determined separately. The quantity of enzyme that lowers the autoxidation rate of pyrogallol is known as one unit of SOD activity.

### 2.4. Protection of Plasmid DNA From Oxidative Damage

EAF and CHF′s ability to prevent DNA damage was evaluated using pUC19 plasmid DNA. To change the supercoiled plasmid DNA into open circular and linear forms caused by AAPH, a modified Zhao and Liu [29] method was used. In this procedure, 1 μg of pUC19 plasmid DNA, 2 μL of 50 mM AAPH, ascorbic acid (100 μg/mL), EAF, and CHF at different dosages (50, 100, and 200 μg/mL) were mixed. After being incubated for 30 min at 37°C, the mixture was electrophoresed on a 1% agarose gel that contained 0.5 μg/mL ethidium bromide. Gel images were captured using a documentation system. Densitometric analysis was performed using ImageJ/Fiji (Version 1.54) on the gel image. Each band area was expressed as a percentage of the total signal within its respective lane.

### 2.5. Assessment of In Vitro Anti‐Inflammatory Properties

The CHF and EAF were evaluated for their anti‐inflammatory potential using the egg albumin denaturation [30] assay and the human RBC (HRBC) membrane stabilization method [31].

#### 2.5.1. HRBC Membrane Stabilization Assay

The HRBC membrane stabilization assay was carried out following the methods of Anosike et al. [32] and Leelaprakash and Dass [33], based on heat‐induced hemolysis [31]. For sample preparation, fresh blood was collected from a healthy volunteer, who had abstained from nonsteroidal anti‐inflammatory drugs (NSAIDs) for at least 2 weeks, into a centrifuge tube containing 2 mL of sodium oxalate to prevent clotting. The sample was stored at 4°C until use and centrifuged at 2500 rpm for 5 min, after which the supernatant was discarded. The packed cell pellet was washed three times with sterile saline (0.9% w/v NaCl) until a clear supernatant was obtained and finally reconstituted with PBS to obtain a 40% (v/v) suspension. For the heat‐induced hemolysis assay, different concentrations (50, 100, 250, and 500 μg/mL) of CHF, EAF, and the standard drug acetylsalicylic acid were mixed with a 10% HRBC suspension in isotonic PBS. The mixtures were incubated at 54°C for 20 min and then centrifuged at 5000 rpm. The absorbance of the supernatant was measured at 540 nm, and the percentage inhibition of hemolysis was calculated to determine membrane stabilization. All samples in the HRBC membrane stabilization assay were run in triplicate, and the results are presented as the mean ± SD of three independent experiments. Using the following formula, the percentage of inhibition of heat‐induced hemolysis was determined:

% inhibition of hemolysis=100×1−OD2−OD1OD3−OD1,

where

OD_1_ = the test sample′s absorbance without heating;
OD_2_ = the heated test sample′s absorbance;
OD_3_ = the heated control sample′s absorbance.

#### 2.5.2. Inhibition of Egg Albumin Denaturation Assay

To evaluate the inhibition of egg albumin denaturation, a reaction mixture of 5 mL was prepared, following the method of Chandra et al. [34]. This mixture contained 0.2 mL of egg albumin (sourced from a fresh hen′s egg) and 2.8 mL of PBS (pH 6.4). It also included 2 mL of EAF and CHF fractions at varying concentrations (50, 100, 200, and 300 μg/mL) in isotonic PBS. Distilled water was used as the control. The mixtures were incubated at 37°C for 15 min and subsequently heated at 70°C for 5 min. After cooling, the absorbance was measured at 660 nm using a spectrophotometer. Aspirin served as the standard at the same concentrations. The percentage of inhibition was calculated using the following formula:

% inhibition of egg albumin denaturation=100×1−OD2−OD1OD3−OD1,

where:

OD_1_ = the test sample′s absorbance without heating;
OD_2_ = the heated test sample′s absorbance;
OD_3_ = the heated control sample′s absorbance.

### 2.6. Chemical Fingerprinting of the Previously Isolated Compound Afzelin

The target compound was previously isolated by column chromatography, whereas its identification and characterization were also confirmed by TLC and NMR spectroscopy. Details have been provided in Akter et al. [21]. TLC profiling was performed using the n‐hexane: ethyl acetate: methanol (1:2:1) solvent system, and spots were visualized under UV light and iodine vapor. The major compound was identified by comparing the *R*_f_ values and spectral characteristics.

Further structural elucidation was conducted using ^1^H and ^13^C NMR spectroscopy (Bruker 400 MHz, CDCl_3_ as the solvent), confirming the presence of kaempferol‐3‐O‐α‐L‐rhamnoside (afzelin) as the principal flavonoid. The spectral data matched previously reported values, and the compound was authenticated based on chemical shifts and coupling patterns. These fingerprinting results are published in Akter et al. [21] and serve as a reference for the current in vivo investigation. Although afzelin was previously isolated and structurally characterized from the EAF, its concentration in the active fractions was not quantitatively determined in the present study.

### 2.7. In Vivo Anti‐Inflammatory Activity Analysis

#### 2.7.1. Carrageenan‐Induced Hind Paw Edema in Mice

The study was conducted in accordance with the guidelines for the care and use of laboratory animals, and the experimental protocol was approved by the University of Rajshahi′s Institutional Animal, Medical Ethics, Biosafety, and Bio‐Security Committee (IAMEBBC, 20 August 2018) under approval number (Approval Memo No 82/320/IAMEBBC/IBSc). All animals were housed under standard laboratory conditions. Mice were kept in iron cages with wood dust bedding, under 28–30°C, 14 h light/10 h dark, and fed a standard pellet diet. All efforts were made to minimize discomfort.

For the experiment, 20 male Swiss albino mice were randomly allocated into four groups (*n* = 5 per group). Animals were fasted overnight with free access to water prior to experimentation. Baseline paw volumes were recorded using slide calipers before treatment, after which the groups received intraperitoneal injections of either 0.9% normal saline, diclofenac sodium (50 mg/kg body weight), and afzelin at doses of 25 and 50 mg/kg body weight. One h following treatment, acute paw edema was induced by subplantar injection of 0.1 mL of 1% carrageenan solution into the right hind paw. Paw volumes were subsequently measured at 1, 2, 3, 4, and 5 h post‐carrageenan administration using slide calipers. The percentage inhibition of paw edema was calculated using the following formula:

% inhibition of edema=mean edema increase in control group−mean edema increase in treated groupmean edema increase in control group×100.

#### 2.7.2. Acute Toxicity Study

The afzelin acute toxicity was evaluated according to the method described by Chinedu et al. [35], with minor modifications to assess acute toxicity and identify suitable doses for subsequent experiments. Fifteen male Swiss albino mice were used, starved for 18 h but allowed water, and divided into five groups of three. They were treated intraperitoneally at doses of 10, 25, 50, 100, and 200 mg/kg body weight. After treatment, the mice were monitored for signs of dullness, nervousness, in coordination, or mortality over 24 h. Based on these results, doses of 25 and 50 mg/kg were selected for further studies.

### 2.8. In Silico Toxicity Prediction for Safety Evaluation of Afzelin

To predict the toxicity of the isolated compound afzelin, we employed ProTox 3.0 [36] and pkCSM [37] servers. ProTox 3.0 (https://tox.charite.de/protox3/) integrates molecular similarity, fragment‐based analysis, and machine learning to forecast various toxicological endpoints, including LD_50_, hepatotoxicity, and carcinogenicity. pkCSM (https://biosig.lab.uq.edu.au/pkcsm/) uses graph‐based signatures to model and predict pharmacokinetic and toxicity profiles, such as hERG inhibition, AMES mutagenicity, and skin sensitization. These tools offer rapid, in silico screening to assess drug safety and guide early‐stage compound evaluation.

### 2.9. In Silico Identification of Afzelin Targets and Inflammatory Regulators

#### 2.9.1. Data Collection and Refinement

The identification of afzelin target proteins and inflammatory regulatory proteins was done through bioinformatics analyses. Using the keyword “inflammation,” inflammatory regulatory proteins were obtained from the NCBI gene database (https://www.ncbi.nlm.nih.gov/) [38] and “filtered for *Homo sapiens.”* The STITCH database (http://stitch.embl.de/) [39] and Swiss Target Prediction [40] were used to determine the direct afzelin target proteins. Venny 2.1 was used to create a Venn diagram that showed the intersections between afzelin′s direct target proteins and inflammatory regulatory proteins (https://bioinfogp.cnb.csic.es/tools/venny/) [41]. Using the STRING [42] database (https://string-db.org/), the interaction of predicted target proteins was built, and the contextual roles of the proteins, particularly their significance in anti‐inflammatory processes, were examined. The PPIN was entered into the Cytoscape program [43] for additional analysis after being exported in the TSV format. Key anti‐inflammatory targets for afzelin were identified by using the CytoHubba software [44] plugin in Cytoscape to rank the proteins according to the degree‐based topological analysis approach.

#### 2.9.2. Ligand Preparation

The three‐dimensional structure of afzelin (PubChem ID: 5316673) was retrieved from PubChem in the SDF format. The structure was then optimized using AutoDock Tools [45] by the addition of polar hydrogen atoms and converted to the PDBQT format.

#### 2.9.3. Preparation of the Target Protein

Crystal structures of the target protein found by bioinformatics analysis were retrieved from the PDB database (http://www.rcsb.org/pdb/). Structures that were derived from *Homo sapiens*, had a resolution of <3 Å, and were bound to a small‐molecule inhibitor were among the inclusion criteria for protein selection. Using AutoDock Tools, the protein was prepared by an optimization process that included removing water and other atoms, adding a polar hydrogen atom, and applying Kollman and Gasteiger charges.

#### 2.9.4. Molecular Docking of Afzelin to the Target Protein

Molecular docking was performed using AutoDock Vina 1.1.2 [46]. To validate the docking protocol, the cocrystallized ligands were redocked into their respective binding sites, and the resulting poses were compared with the experimentally determined ligand conformations using root mean square deviation (RMSD). RMSD values ≤ 2.0 Å were considered indicative of acceptable reproduction of the crystallographic ligand pose. The docking search space was defined separately for each target protein based on the corresponding cocrystallized ligand‐binding site. The docking parameters, including the search box center and dimensions, are summarized in Table 1. Binding scores were recorded for each ligand, and molecular interactions were visualized using PyMOL [47] and BIOVIA Discovery Studio 2024 [48].

**Table 1:** Validation results of molecular docking for native ligands with target proteins.

Protein	PDB ID	Search‐box dimensions (Å)	Center coordinates (*X*, *Y*, *Z*)	RMSD (Å)
TNF	2AZ5	18.75 × 15.75 × 15.00	−21.716, 69.751, 39.208	1.82
PTGS2	5IKR	16.50 × 16.50 × 18.75	37.904, 2.682, 58.901	0.60
HSP90AA1	2XJX	21.00 × 21.00 × 20.25	13.746, −2.890, 13.229	1.95
HSP90AB1	3NMQ	21.75 × 16.50 × 19.50	0.956, 11.945, 27.893	0.75
PI3K	7R9V	23.25 × 25.50 × 22.50	−18.889, 10.306, 28.290	1.78
ABL1	4WA9	18.75 × 18.75 × 21.00	12.791, 153.508, 35.849	1.45
RPS6KB1	3WF7	21.75 × 21.00 × 15.75	12.356, −9.263, 11.620	0.80
JAK3	3PJC	18.75 × 21.75 × 22.50	8.308, −4.109, 8.074	1.86
AKT2	3D0E	15.75 × 18.00 × 27.75	21.999, −16.285, 8.284	0.06
IL‐2	1PW6	21.00 × 16.50 × 18.75	88.199, 25.616, 16.878	0.84
PDGFRA	5GRN	23.25 × 22.50 × 22.50	−12.327, 2.485, −10.698	2.10

### 2.10. Ethical Approval

Blood samples were collected from a healthy adult volunteer (willingly donated) under institutional ethical approval. Ethical clearance for both human blood collection and animal experiments was obtained from the IBSc, University of Rajshahi’s IAMEBBC for Experiments on animal, human, microbes, and living natural sources under Approval Memo Number 82/320/IAMEBBC/IBSc, dated 20 August 2018. The protocol for using human blood cells and mice in anti‐inflammatory research was approved by this committee, and written informed consent was obtained from the participant prior to sample collection.

### 2.11. Statistical Analysis

All experimental data were expressed as the mean ± SD. Statistical analysis was performed using the IBM SPSS Statistics version 20.0. Differences among the experimental groups were analyzed using one‐way analysis of variance (ANOVA), followed by the Dunnett’s multiple‐comparison post hoc test.

## 3. Results

### 3.1. Ex Vivo Antioxidant Activities

#### 3.1.1. Protection of Oxidative Damage of Erythrocytes Induced by AAPH

The oxidative damage induced by AAPH in human erythrocytes and the protective effects of CHF and EAF are shown in Figure 1. When erythrocytes were incubated as a 5% hematocrit suspension in PBS at 37°C in air, they remained stable, with minimal hemolysis observed over 6 h (11.23% ± 0.83%). However, upon addition of the water‐soluble radical initiator AAPH (50 mM), hemolysis increased in a time‐dependent manner. The reduced hemolysis observed in the presence of CHF and EAF was consistent with protection against AAPH‐induced oxidative damage.

**Figure 1:**
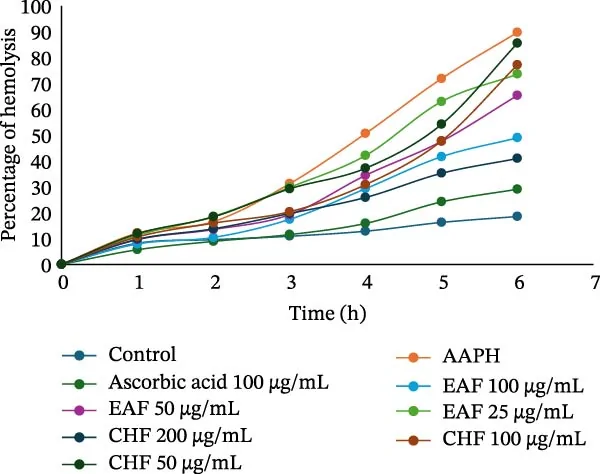
Effects of EAF and CHF on erythrocyte oxidative damage caused by AAPH. The values are given as the three experiments′ mean ± SD.

Specifically, CHF (at concentrations of 50, 100, and 200 μg/mL) and EAF (at 25, 50, and 100 μg/mL) suppressed AAPH‐induced hemolysis in a concentration‐ and time‐dependent fashion, as detailed in Figure 1. Different concentration ranges were used for CHF and EAF because their distinct chemical compositions and potencies require varied doses to effectively demonstrate their protective effects.

#### 3.1.2. Measurement of Lipid Peroxidation of Erythrocytes

AAPH‐induced lipid peroxidation in erythrocytes was evaluated by monitoring MDA formation in the presence of CHF and EAF of *P. dulce* (Figure 2). In the untreated control group, MDA levels were 2.06 ± 0.27 pmol/g Hb, 2.27 ± 0.29 pmol/g Hb, and 2.87 ± 0.15 pmol/g Hb at 2 h, 4 h, and 6 h, respectively. Incubation with 50 mM AAPH significantly increased MDA levels in a time‐dependent manner, reaching 8.71 ± 0.34, 14.90 ± 0.25, and 19.02 ± 0.57 pmol/g Hb at the corresponding time points, thereby confirming that AAPH induces progressive lipid peroxidation in erythrocytes. Pretreatment with CHF and EAF fractions markedly inhibited AAPH‐induced MDA formation in a concentration‐dependent manner. For consistency, EAF was also evaluated at 200 μg/mL, allowing comparison with CHF at the same concentration to assess their relative protective effects.

**Figure 2:**
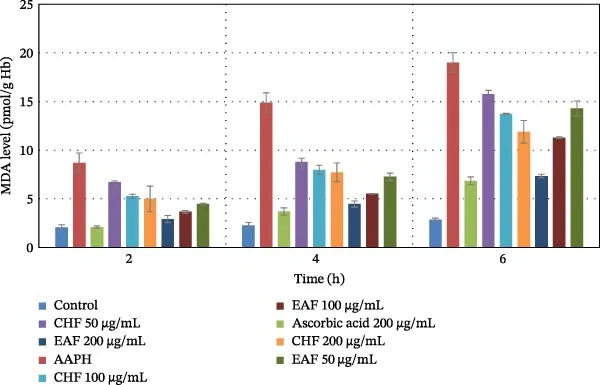
Effects of CHF and EAF on AAPH‐induced alterations in erythrocyte MDA levels. Either PBS (control) or CHF and EAF extracts at the indicated concentrations were preincubated for 30 min with erythrocyte suspensions (5% hematocrit), followed by incubation with 50 mM AAPH at 37°C for 2, 4, and 6 h. MDA levels were expressed as pmol/g Hb. Data represent the mean ± SD of three independent experiments.

#### 3.1.3. Effects on AAPH‐Induced Changes in Erythrocyte GSH Content

The AAPH‐induced alterations in erythrocyte GSH levels in the presence of CHF and EAF extracts of *P. dulce* are presented in Figure 3. In the control group, erythrocyte GSH levels were 4.70 ± 0.10 µmol/g Hb, 3.68 ± 0.12 µmol/g Hb, and 2.73 ± 0.23 µmol/g Hb at 2 h, 4 h, and 6 h, respectively. Incubation with 50 mM AAPH resulted in a significant, time‐dependent depletion of cytosolic GSH, with progressively reduced levels at the same time intervals. Notably, treatment with CHF and EAF extracts of *P. dulce* effectively attenuated AAPH‐induced GSH depletion, thereby preserving the intracellular antioxidant status.

**Figure 3:**
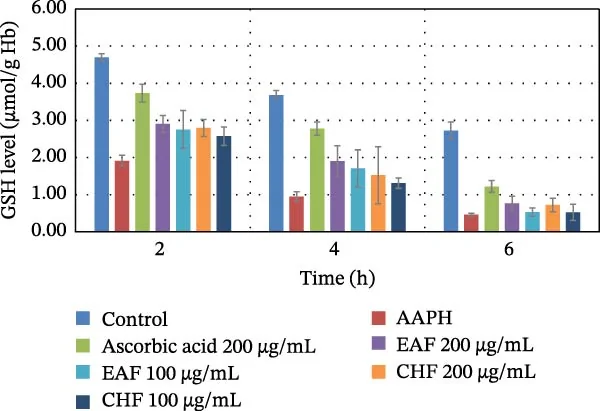
Effects of EAF and CHF on AAPH‐induced alterations in erythrocyte GSH levels. Either PBS (control) or CHF and EAF extracts at the specified concentrations were preincubated for 30 min with erythrocyte suspension at 5% hematocrit. It was then incubated at 37°C for 2, 4, and 6 h with 50 mM AAPH. The mean ± SD of the three experiments was used to express the values.

#### 3.1.4. Effects on AAPH‐Induced Changes in Erythrocyte SOD Activity

Effects of CHF and EAF of *P. dulce* on AAPH‐induced changes in erythrocyte SOD activity are shown in Figure 4. In the control group, erythrocyte SOD activity was 0.29 ± 0.02 U/mg Hb, 0.21 ± 0.02 U/mg Hb, and 0.11 ± 0.02 U/mg Hb at 2 h, 4 h, and 6 h, respectively. Incubation with 50 mM AAPH caused a significant time‐dependent decrease in SOD activity at these time points. Both CHF and EAF extracts effectively prevented the AAPH‐induced decline in erythrocyte SOD activity, as illustrated in Figure 4. Furthermore, when erythrocytes were incubated with CHF and EAF extracts alone (at 200 and 100 μg/mL), SOD activity remained stable at levels comparable to those in the untreated control samples.

**Figure 4:**
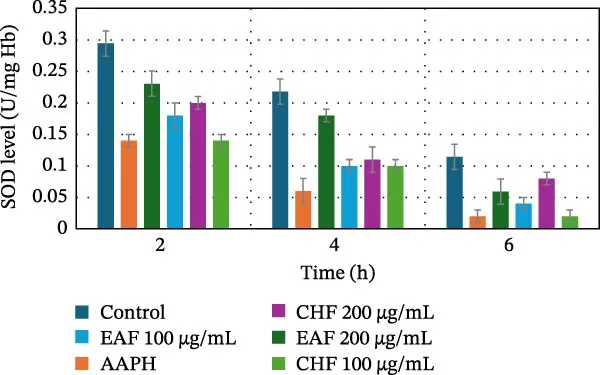
Effects of CHF and EAF of *P. dulce* on AAPH‐induced changes in erythrocyte SOD activity. Erythrocyte suspension at 5% hematocrit was incubated with PBS (control), or preincubated with CHF and EAF at the indicated concentrations for 30 min. Then it was incubated with 50 mM AAPH at 2, 4, and 6 h at 37°C. Values were expressed as mean ± SD of three experiments.

#### 3.1.5. Protective Effects on DNA Oxidative Damage

The protective effects of the CHF and EAF of *P. dulce* leaves against AAPH‐induced oxidative DNA damage were evaluated using the pUC19 plasmid model (Figure 5), and the corresponding original gel image is provided in Figure S1. Exposure to 50 mM AAPH resulted in extensive DNA degradation, as evidenced by the DNA pattern in lane 2. In contrast, treatment with CHF and EAF fractions at different concentrations (50, 100, and 200 µg/mL) resulted in increased retention of DNA bands, particularly band 3, indicating a protective effect against AAPH‐induced oxidative DNA damage.

**Figure 5:**
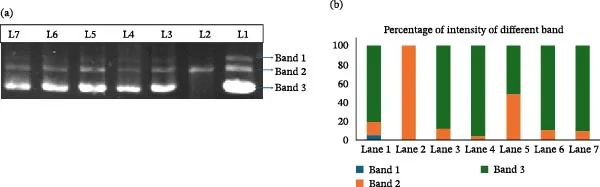
Protective effect of EAF and CHF on pUC19 plasmid DNA damage induced by AAPH. (a) lane 1: native DNA; lane 2: DNA + AAPH; lane 3: DNA + AAPH + 100 μg EAF; lane 4: DNA + AAPH + 50 μg EAF; lane 5: DNA + AAPH + 100 μg ascorbic acid; lane 6: DNA + AAPH + 200 μg CHF; lane 7: DNA + AAPH + 100 μg CHF. (b) Densitometric quantification of DNA band intensity (%) for each lane expressed as a percentage of total lane signal.

Densitometric analysis was performed using ImageJ/Fiji (Version 1.54) [49] (analyze > gels) on the single gel, as shown in Figure 5a. The area of each band was expressed as a percentage of the total signal within its respective lane (% of lane total = band area in each lane × 100). Because the densitometric analysis was based on a single gel, the percentage of intensities of each band was presented descriptively in Table S1, and no formal statistical analysis was performed.

In the AAPH‐treated control (lane 2), only band 2 was detectable and therefore accounted for 100% of the total lane signal. In contrast, band 3 was retained in the CHF‐ and EAF‐treated samples and represented the predominant DNA band in most treatment lanes. Therefore, band 3 was selected as the representative band for illustrating the quantitative trend in DNA protection, as shown in Figure 5b. Band 1 was detectable in lane 1 but was not consistently observed across the treated lanes and was therefore not used as a representative measure of DNA protection.

### 3.2. Evaluation of In Vitro Anti‐Inflammatory Activity

#### 3.2.1. Heat‐Induced Hemolysis

The result of anti‐inflammatory activity was determined by the heat‐induced RBC membrane stabilization method. The test results are shown in Figure 6. The EAF and CHF fractions of *P. dulce* leaves and ASA at concentrations of 50, 100, 250, and 500 µg/mL showed a concentration‐dependent anti‐inflammatory activity, and the percentage protection of RBC membrane hemolysis increased with increased concentration of samples. At a concentration of 500 µg/mL EAF, CHF showed 57.31% ± 0.80% and 51.39% ± 0.27% inhibition of RBC hemolysis, respectively, compared with 80.56% ± 0.37% inhibition produced by standard ASA at 500 µg/mL. The results obtained in this experiment indicated that EAF shows significant anti‐inflammatory activity.

**Figure 6:**
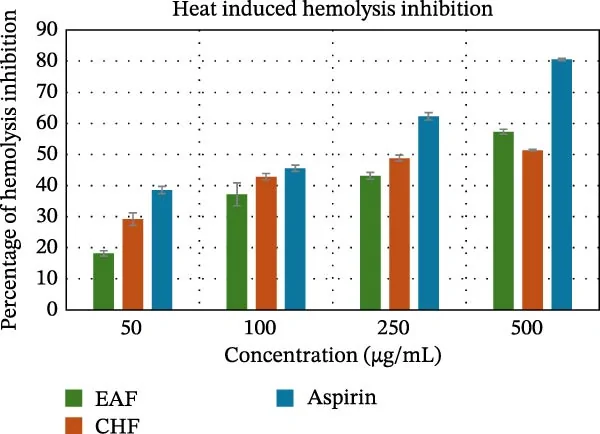
Heat‐induced hemolysis inhibition by EAF, CHF, and aspirin at varying concentrations. EAF and CHF exhibited dose‐dependent protective effects against hemolysis, with aspirin showing the highest inhibition across all concentrations. Error bars represent standard deviation, indicating reproducibility across replicates. Data suggest comparative efficacy of test fractions in stabilizing erythrocyte membranes under thermal stress.

#### 3.2.2. Egg Albumin Denaturation Method

The result of the anti‐inflammatory activity was determined by the egg albumin denaturation method. The test results are shown in Figure 7. The EAF and CHF fractions of *P. dulce* leaves with ASA, at concentrations of 50, 100, 200, and 300 µg/mL, showed a concentration‐dependent anti‐inflammatory activity, and the percentage protection of albumin denaturation increased with increased concentration of samples. At a concentration of 300 µg/mL EAF, CHF exhibited 39.74% ± 0.77% and 37.20% ± 2.16% inhibition of albumin denaturation, respectively, compared with 61.52% ± 0.58% inhibition produced by standard ASA at 300 µg/mL.

**Figure 7:**
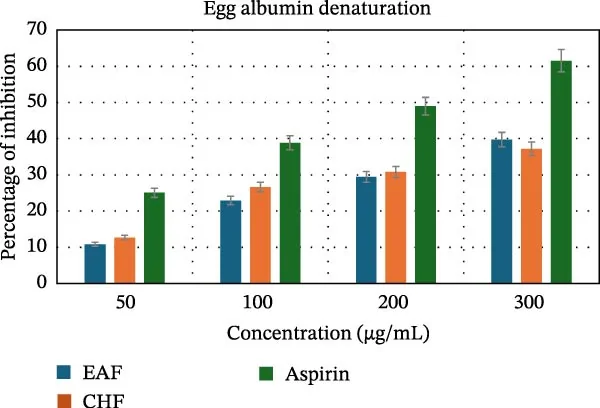
Percent inhibition of egg albumin denaturation by EAF, CHF, and aspirin. All test samples demonstrated a concentration‐dependent increase in inhibition, with aspirin consistently exhibiting the highest activity, followed by EAF and CHF. Error bars represent standard deviation, indicating reproducibility across replicates. These results suggest potential anti‐inflammatory properties of the test fractions through protein stabilization under denaturing conditions.

### 3.3. Evaluation of In Vivo Anti‐Inflammatory Activity of Afzelin

Afzelin (kaempferol‐3‐O‐α‐L‐rhamnoside), previously isolated from the EAF of *P. dulce* leaves, was characterized and reported in our earlier publication [21].

#### 3.3.1. In Silico Toxicity Prediction of Afzelin

The in silico toxicity profile of afzelin was evaluated using ProTox 3.0 and pkCSM. According to ProTox 3.0, afzelin exhibited a predicted oral LD_50_ value of 5000 mg/kg body weight, corresponding to toxicity class 5, which indicates relatively low predicted acute oral toxicity. The predicted dose value was 2319.9 mg/kg, compared with a dataset average of 3500 mg/kg (Figure 8). These predictions provide an initial computational assessment of the acute toxicity profile of afzelin.

**Figure 8:**
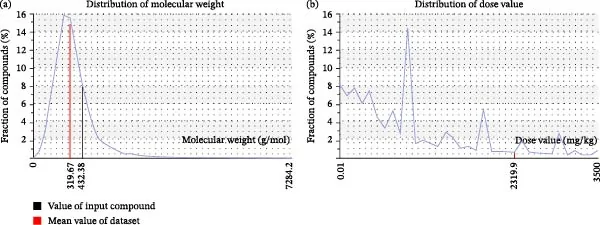
Comparative distribution of molecular weight and dose values for afzelin [36]. (a) The distribution of molecular weights (g/mol) across the dataset, with fraction (%) plotted on the *y*‐axis. (b) The distribution of dose values (mg/kg), showing a nonuniform pattern with multiple peaks. These visualizations contextualize the physicochemical and pharmacological positioning of the afzelin relative to the broader dataset.

Additional toxicity‐ and safety‐related properties were predicted using the pkCSM platform. pkCSM predicted afzelin to be nonmutagenic (AMES toxicity: no), nonhepatotoxic, and nonsensitizing to the skin. It was predicted to inhibit hERG II but not hERG I, indicating that potential cardiac safety concerns may warrant further experimental evaluation. The predicted oral rat acute toxicity (LD_50_) and chronic toxicity (LOAEL) values were 2.61 mol/kg and 2.799 log mg/kg body weight/day, respectively. Additional pkCSM‐predicted toxicity endpoints are presented in Table 2. These computational predictions should be considered preliminary and require experimental validation.

**Table 2:** ProTox 3.0‐ and pkCSM‐predicted toxicity and safety profile of afzelin.

Prediction platform	Parameter	Predicted value
ProTox 3.0	Oral LD_50_	5000 mg/kg
ProTox 3.0	Toxicity class	5
pkCSM	AMES toxicity	No
pkCSM	hERG I inhibitor	No
pkCSM	hERG II inhibitor	Yes
pkCSM	Oral rat acute toxicity (LD50)	2.61 mol/kg
pkCSM	Oral rat chronic toxicity (LOAEL)	2.799 log mg/kg body weight/day
pkCSM	Hepatotoxicity	No
pkCSM	Skin sensitization	No
pkCSM	*T. pyriformis* toxicity	0.285 log µg/L
pkCSM	Minnow toxicity	3.726 log mM
pkCSM	Cytotoxicity	0.93 (inactive)

#### 3.3.2. Evaluation of Carrageenan‐Induced Paw Edema

All experimental animals exhibited a time‐dependent increase in paw edema following carrageenan administration (Table 3; Figure 9). In contrast, the treated groups showed a dose‐dependent reduction in edema. At 5 h after carrageenan administration, diclofenac‐Na (50 mg/kg) produced a 70.83% inhibition of paw edema. Among the test groups, afzelin at 50 mg/kg body weight produced the greatest inhibition (56.16%), followed by afzelin at 25 mg/kg (38.56%). Although the inhibitory effect of afzelin at 50 mg/kg was lower than that of diclofenac‐Na, the findings demonstrate a dose‐related anti‐inflammatory effect of afzelin in the carrageenan‐induced paw edema model.

**Table 3:** Inhibitory effect of afzelin and diclofenac‐Na on carrageenan induced paw edema in mice.

Sample	Percentage of inhibition of edema				
	1 h	2 h	3 h	4 h	5 h
Control					
Afzelin 25 mg/kg BW	34.88 ± 0.4^∗^	31.63 ± 0.13^∗^	33.52 ± 0.71^∗^	33.90 ± 0.32^∗^	38.56 ± 0.03^∗^
Afzelin 50 mg/kg BW	45.50 ± 0.05^∗^	38.24 ± 0.09^∗^	41.97 ± 0.63^∗^	42.69 ± 0.28^∗^	56.16 ± 0.37^∗^
Diclofenac‐Na 50 mg/kg BW	55.76 ± 0.07	50.06 ± 0.08	49.97 ± 0.79	63.81 ± 0.36	70.83 ± 0.83

*Note:* Data are expressed as the mean ± SD. Analysis of variance followed by Dunnett′s post hoc test (IBM‐SPSS/20).  ^∗^ Indicates a statistically significant difference compared with the standard drug‐treated group (*p* < 0.05).

**Figure 9:**
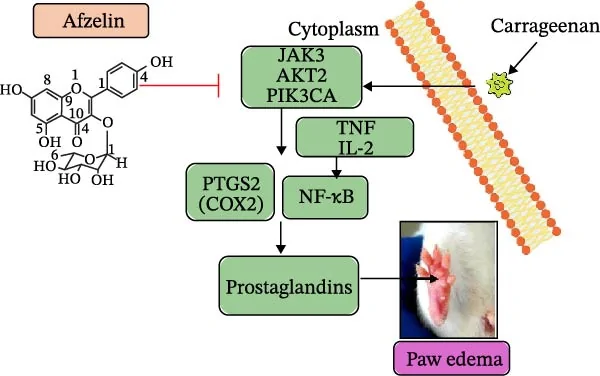
Proposed mechanism illustrating potential anti‐inflammatory pathways through which afzelin may exert its activity based on in vivo findings and computational predictions. Carrageenan stimulates JAK3, PIK3CA, and AKT2 to act on NF‐κB. This perpetuates COX‐2 (PTGS2) and prostaglandin production, while TNF and IL‐2 further increase NF‐κB activity and exacerbate inflammation. Afzelin disrupts these processes decreasing cytokine signaling, NF‐κB activation, and prostaglandin production ultimately resolving paw edema.

### 3.4. Identification of Afzelin′s Anti‐Inflammatory Protein Targets

To elucidate the molecular mechanisms underlying the anti‐inflammatory activity of afzelin, a network pharmacology approach was employed to identify its potential therapeutic targets. A total of 2648 inflammation‐associated human genes were retrieved from the NCBI gene database. The STITCH database identified 10 proteins as direct targets of afzelin, namely, SGK1, SGK2, RPL10A, RPS6KB1, AKT2, RPS6KB2, RPS6KA1, RPS6KA3, and RPS6KA6 (Figure 10a). In contrast, Swiss Target Prediction identified 100 potential protein targets for afzelin (Figure 10b). After removing duplicate entries, the predicted targets from both databases were combined and compared with the inflammation‐associated genes using Venny 2.1 (Figure 10c).

**Figure 10:**
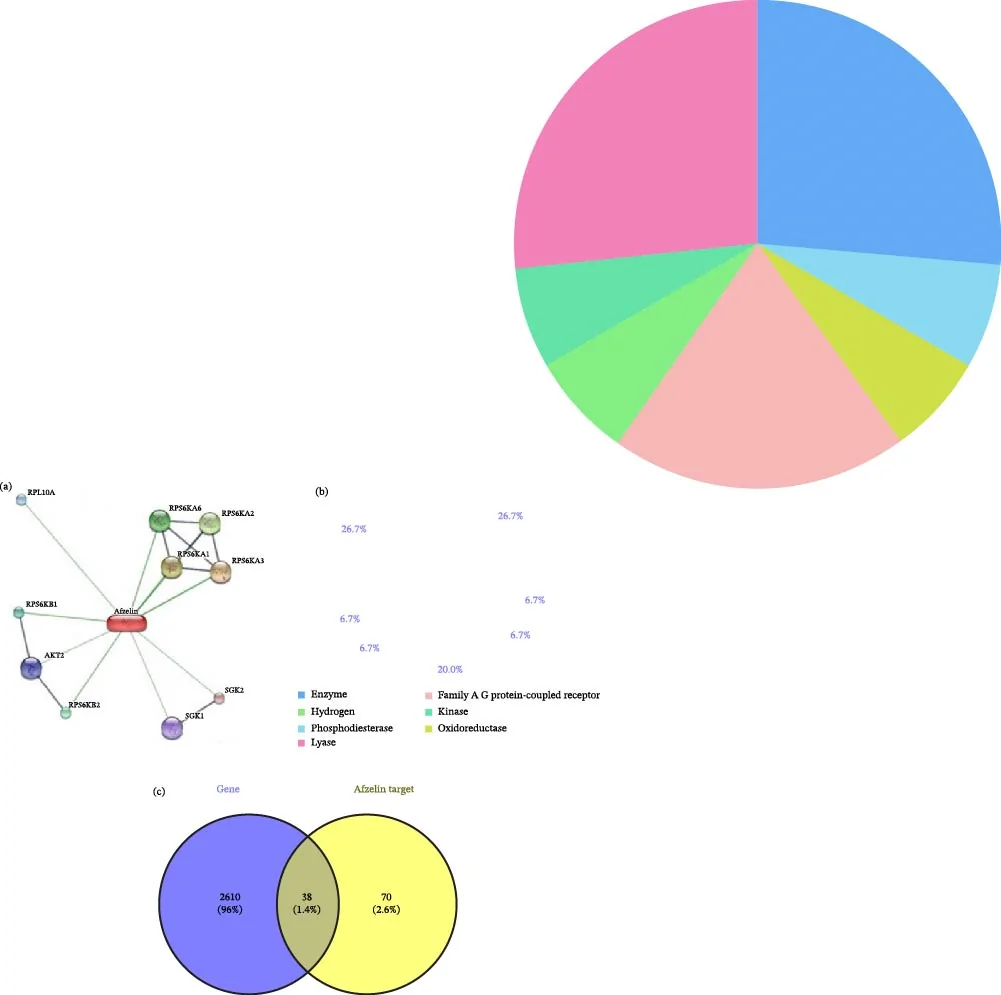
Integrated visualization of afzelin‐associated molecular targets and gene overlap. (a) Direct target protein generated using STITCH and Swiss target prediction, (b) pie chart classifies afzelin targets, and (c) Venn diagram shows 38 overlapping genes with therapeutic relevance.

This analysis identified 38 overlapping proteins, which were considered the potential anti‐inflammatory targets of afzelin. The 38 overlapping proteins were imported into STRING to construct a protein–protein interaction (PPI) network and investigate their functional relationships (Figure 11a).

**Figure 11:**
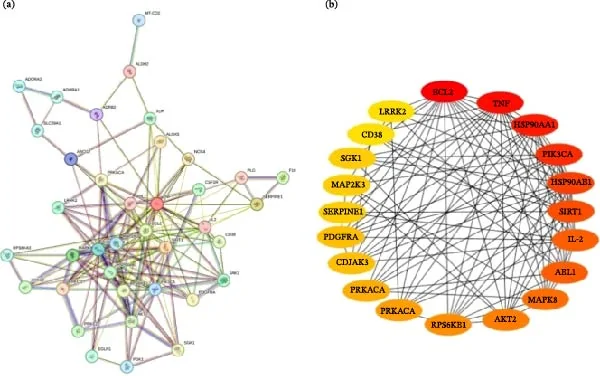
Protein–protein interaction networks of afzelin‐associated targets. (a) STRING‐based network of afzelin‐related proteins showing interaction types; (b) focused subnetwork highlighting key signaling targets by interaction relevance.

However, the 20 proteins with the highest degree values were selected based on their degree of interaction. Their interaction network is shown in Figure 11b, and the degree values are listed in Table 4. Among these, six proteins exhibited anti‐inflammatory functions (SIRT1, IL‐2, AKT2, PRKACA, CD38, and MAP2K3), while 12 proteins displayed proinflammatory roles (TNF, PTGS2, HSP90AA1, HSP90AB1, PI3K, PRKCD, ABL1, RPS6KB1, JAK3, PDGFRA, SERPINE1, and LRRK2). BCL2 and MAPK8 were not assigned to either functional category in the present analysis. For structural studies, crystallographic data were retrieved for selected targets, applying the criteria of no mutations, resolution <3 Å, and complexed with inhibitory molecules. Based on these filters, eight proinflammatory proteins and 3 anti‐inflammatory proteins (IL‐2, AKT2, and PRKACA) with their native ligands were chosen for further molecular docking and binding analysis.

**Table 4:** List of top 20 afzelin target protein according to the degree score.

Rank	Name	Score	Rank	Name	Score
1	BCL2	24	11	RPS6KB1	11
2	TNF	23	12	PRKACA	10
3	HSP90AA1	20	13	PRKCD	9
4	PI3K	18	14	JAK3	8
5	HSP90AB1	17	15	PDGFRA	8
6	SIRT1	15	16	SERPINE1	7
7	IL‐2	14	17	MAP2K3	7
8	ABL1	14	18	SGK1	7
9	MAPK8	13	19	CD38	6
10	AKT2	12	20	LRRK2	6

### 3.5. Molecular Docking Studies

The docking protocol was validated by redocking the cocrystallized native ligands into their respective binding sites. Most target proteins showed RMSD values ≤2.0 Å, indicating acceptable reproduction of the experimentally determined ligand poses. PDGFRA showed a slightly higher RMSD of 2.1 Å, and the corresponding docked pose should therefore be interpreted with caution. The grid spacing was set at 0.375 Å, while the target‐specific search box dimensions and center coordinates are presented in Table 1.

Afzelin exhibited negative predicted docking scores against all evaluated target proteins, indicating favorable predicted interactions within the selected binding sites (Table 5). Among the evaluated targets, afzelin showed its most favorable predicted docking score against JAK3 (−9.60 kcal/mol), whereas the least favorable score was observed for IL‐2 (−5.90 kcal/mol).

**Table 5:** Binding energy analysis of afzelin with target protein through molecular docking.

Protein	PDB ID	Inhibitor bound	Binding energy (kcal/mol)	
			Native ligand	Afzelin
TNF	2AZ5	307 (small molecule inhibitor)	−5.5	−6.7
PTGS2	5IKR	Mefenamic acid	−8.4	−8.1
HSP90AA1	2XJX	Onalespib	−9.4	−8.2
HSP90AB1	3NMQ	EC144	−8.9	−9.4
PI3K	7R9V	Covalent inhibitor 19	−11.1	−9.2
ABL1	4WA9	Axitinib	−8.5	−7.4
RPS6KB1	3WF7	ATP analogs (FS9)	−8.6	−8.8
JAK3	3PJC	Selective ATP‐site inhibitor	−10.3	−9.6
AKT2	3D0E	G93	−10.2	−8.3
IL‐2	1PW6	FRB	−7.7	−5.9
PDGFRA	5GRN	WQ‐C‐159	−10.6	−7.9

Afzelin exhibited more favorable predicted docking scores than the corresponding cocrystallized ligands for TNF (2AZ5) and HSP90AB1 (3NMQ), whereas less favorable scores were observed for PTGS2 (5IKR), HSP90AA1 (2XJX), PI3K (7R9V), ABL1 (4WA9), JAK3 (3PJC), AKT2 (3D0E), IL‐2 (1PW6), and PDGFRA (5GRN). For RPS6KB1 (3WF7), the predicted docking score of afzelin (−8.80 kcal/mol) was slightly more favorable than that of the cocrystallized ligand (−8.60 kcal/mol).

Representative interaction profiles of afzelin and the corresponding cocrystallized ligands with selected target proteins are presented in Figures 12–15. Afzelin was predicted to form hydrogen‐bonding, hydrophobic, and π‐related interactions with residues within the respective binding sites. These predicted interactions included residues such as HIS388, TYR385, ASN384, THR212, and HIS207 in PTGS2; TYR119 and TYR151 in TNF; GLU849 in PI3K; and ASP236 in RPS6KB1.

**Figure 12:**
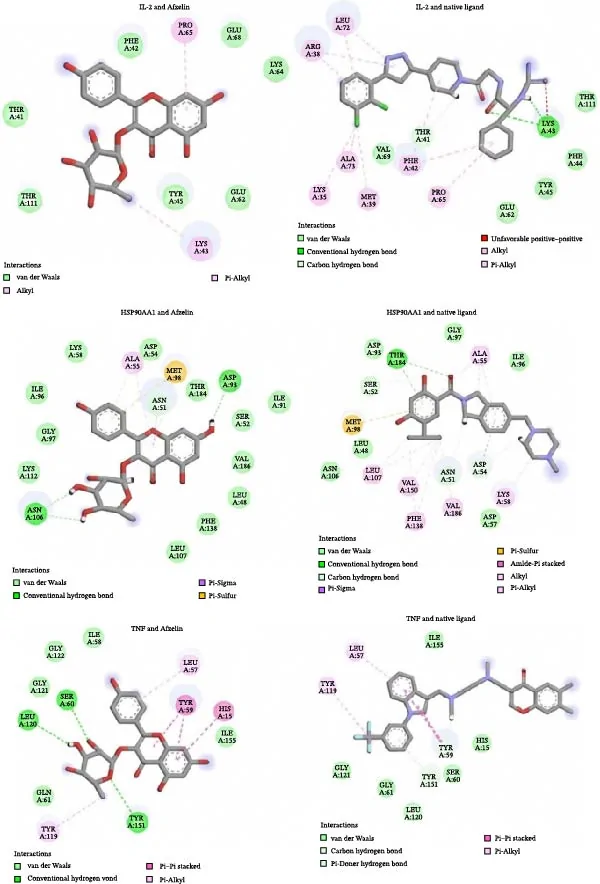
2D diagram of the interaction analysis of IL‐2, HSP90AA1, and TNF with afzelin and native ligands.

**Figure 13:**
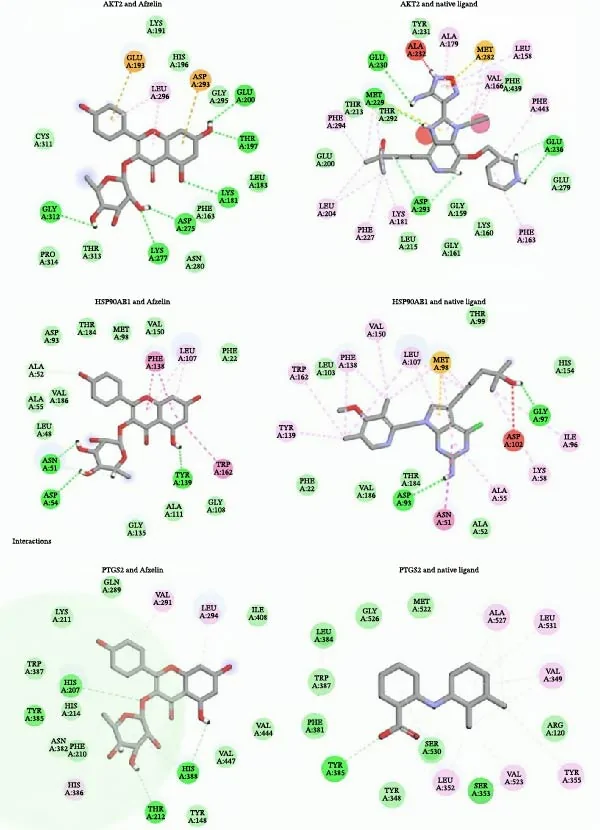
2D diagram of the interaction analysis of AKT2, HSP90AB1, and PTGS2 with afzelin and native ligands.

**Figure 14:**
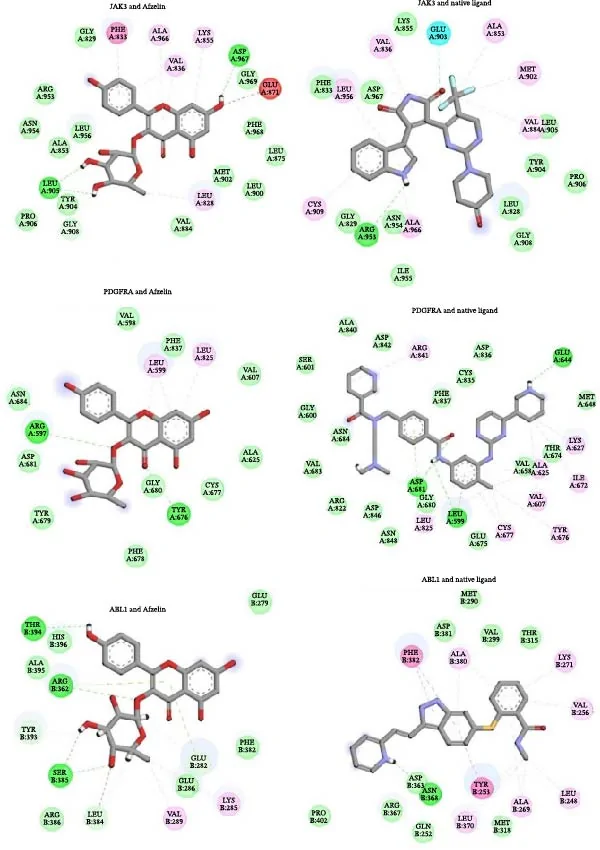
2D diagram of the interaction analysis of JAK3, PDGFRA, and ABL1 with afzelin and native ligands.

**Figure 15:**
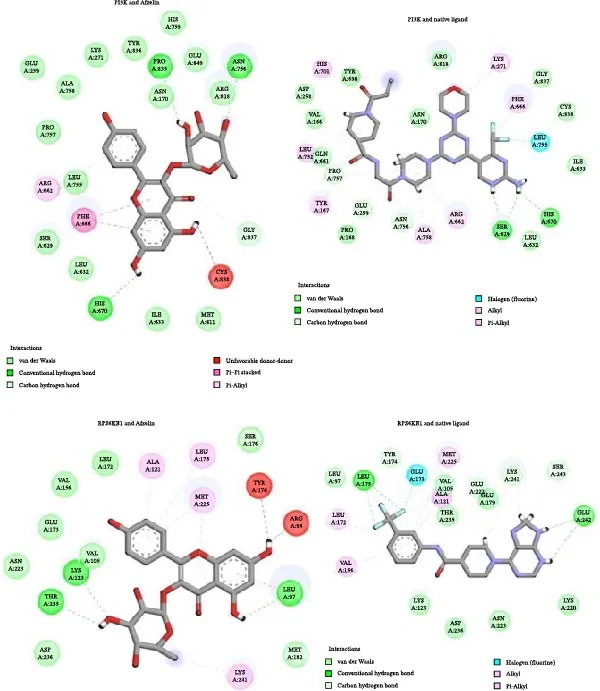
2D diagram of the interaction analysis of PI3K and RPS6KB1 with afzelin and native ligands.

Within JAK3, afzelin was predicted to interact with PHE833 and TYR904 through aromatic interactions and with ASP967 through a conventional hydrogen bond. Additional hydrophobic contacts involving ALA966, VAL836, and LEU828 were also predicted. An unfavorable acceptor–acceptor interaction involving GLU871 was additionally observed. These computationally predicted interactions provide hypotheses regarding possible interactions of afzelin with inflammation‐associated targets and require experimental validation.

## 4. Discussion

In the present study, *P. dulce* leaf fractions were systematically investigated through integrated ex vivo antioxidant assays, DNA protection studies, in vitro and in vivo anti‐inflammatory evaluations, and molecular docking analysis to elucidate the pharmacological potential of afzelin isolated from the EAF. The collective findings provide scientific evidence supporting the traditional medicinal use of *P. dulce* and suggest that afzelin, a major flavonoid glycoside, is important for the plant’s biological activity.

Oxidative stress is a pivotal factor in the initiation of DNA damage and inflammation [24, 25, 50]. In our study, the extracts showed antioxidant effects, which correlated with their phytochemical composition, particularly the presence of flavonoids and phenolic compounds. This is consistent with previous research showing that *P. dulce* extracts rich in polyphenols are effective in scavenging radicals and chelating metals [21]. Importantly, the fractions also provided marked protection against oxidative DNA damage, suggesting their potential role in maintaining genomic stability. This DNA‐protective property agrees with prior studies where afzelin and related flavonoids reduced genotoxic lesions and enhanced cellular resilience under oxidative stress [51].

In the present investigation, the quantification of MDA and related oxidative stress markers was expressed relative to Hb content to ensure accuracy and comparability among samples. Normalizing to Hb minimizes variation caused by differences in the erythrocyte number and hematocrit, providing a reliable internal standard for evaluating lipid peroxidation and antioxidant enzyme activities. This approach is widely used in erythrocyte‐based assays because Hb concentration reflects the total cellular protein content and ensures consistent representation of oxidative status [24, 29].

Initial experiments with the EAF and CHF fractions evaluated the in vitro anti‐inflammatory activity of the leaves and showed the highest response by EAF and CHF in protecting HRBCs from membrane hemolysis and egg albumin from denaturation. Since most research has employed in vitro protection of HRBCs and proteins as a means of assessing anti‐inflammatory activity, this result was promising.

Although the membrane stabilization and protein denaturation assays are indirect methods, they are widely recognized as valuable preliminary tools for evaluating anti‐inflammatory potential. The RBC membrane is structurally comparable to the lysosomal membrane, both of which are vulnerable to oxidative damage by free radicals. Lysosomal membrane destabilization leads to the release of hydrolytic enzymes, such as proteases and phosphatases, resulting in cellular injury, inflammation, and apoptosis. Since the erythrocyte membrane shares structural and compositional similarities with the lysosomal membrane, the ability of plant extracts to prevent hemolysis in RBCs indicates the potential to stabilize lysosomal membranes and inhibit the release of inflammatory mediators [30–32]. Therefore, membrane stabilization reflects the ability of extracts/compounds to protect erythrocyte and lysosomal membranes from stress‐induced lysis, thereby preventing the release of inflammatory mediators. Similarly, inhibition of protein denaturation indicates the capacity to maintain the protein structure under stress conditions, an event analogous to suppression of inflammation‐related protein unfolding in vivo. Several studies have validated the predictive value of these assays for screening plant‐derived anti‐inflammatory agents [30–32]. Thus, the positive outcomes observed for *P. dulce* fractions suggest that their anti‐inflammatory effects may involve both antioxidant and membrane‐protective mechanisms.

Therefore, the most active fraction, EAF, from which afzelin was isolated, was selected for further detailed investigation of its specific effects in the in vivo carrageenan‐induced paw edema model. The *in vivo* anti‐inflammatory evaluations further revealed that *P. dulce* fractions, particularly the EAF from which afzelin was previously isolated, suppressed acute inflammation in experimental models. Afzelin may exert anti‐inflammatory effects through modulation of TNF, IL‐2, PTGS2, JAK3, AKT2, and related signaling pathways, consistent with previous reports describing afzelin’s inhibitory effects on inflammatory responses [18, 52]. Additionally, afzelin has also been reported to reduce C. acnes‐induced inflammation in sebocytes [18] and to attenuate NF‐κB and MAPK signaling pathways, which supports our present findings.

Although a few studies [53, 54] have explored the anti‐inflammatory effects of *P. dulce* plant extracts in carrageenan‐induced paw edema models, no studies have directly examined afzelin’s role in this context.

Our molecular‐docking analysis complemented the experimental findings by identifying potential molecular interactions between afzelin and inflammation‐related and cytokine mediators. Afzelin formed hydrogen bonds, hydrophobic contacts, and aromatic stacking interactions with critical residues, often overlapping with native ligand‐binding sites. This aligns with recent docking work that found afzelin’s potential to target RPS6KA1 in leukemia models [55]. The binding profiles observed here underscore afzelin’s multi‐target anti‐inflammatory potential.

Furthermore, although afzelin has been recognized for its antioxidant and anti‐inflammatory properties, research in this area remains limited [56], particularly regarding its molecular interactions with key inflammatory proteins. Most available studies rely on general phytochemical screenings or isolated in vitro assays, and only a few *in silico* investigations have explored its binding behavior with targets such as TNF, COX‐2, JAK3, or AKT2. Even those computational studies often lack experimental follow‐up. To bridge this gap, we carried out both in vivo experiments using the carrageenan‐induced paw edema model, a well‐established method for evaluating acute inflammation, and in silico docking analyses. This dual approach allowed us to directly observe afzelin’s effects in a physiological setting, offering new insights into its therapeutic relevance and helping validate its role beyond theoretical predictions.

Overall, our results show that *P. dulce* leaf fractions, particularly the isolated compound afzelin, could be valuable sources of anti‐inflammatory agents with broad activity. Combining biochemical in vivo and computational approaches provided a comprehensive understanding of the plant’s pharmacological mechanisms. Because afzelin was predicted to interact with multiple inflammatory targets and exhibit activity across several test systems, it may serve as a promising lead molecule for developing new anti‐inflammatory drugs beneficial for conditions driven by oxidative stress and immune dysregulation.

## 5. Limitations, Future Directions, and Conclusion

While the present study provides valuable insights into the antioxidant and anti‐inflammatory potential of *P. dulce* leaf fractions and the possible molecular interactions of afzelin with inflammatory targets such as JAK3, AKT2, COX‐2, IL‐2, HSP90AB1, and PI3K, several limitations should be acknowledged. Although afzelin was previously isolated and structurally characterized from the EAF, its concentration was not quantitatively determined in the active fractions used in the present study. Therefore, the observed biological activities of the fractions cannot be attributed exclusively to afzelin, and the potential contributions of other phytoconstituents cannot be excluded. Future studies employing quantitative analytical techniques such as HPLC or LC–MS are needed to correlate the afzelin content with biological activity.

In addition, although molecular docking analyses suggested interactions of afzelin with several inflammation‐related proteins, these computational findings represent predicted molecular mechanisms. Experimental validation using target‐specific biochemical assays, enzyme inhibition studies, gene and protein expression analyses, or pathway‐based investigations will be necessary to confirm these proposed mechanisms of action. Furthermore, although promising binding affinities were observed, afzelin exhibited a lower binding affinity than the native ligands for several target proteins, indicating opportunities for structural optimization to improve potency and target selectivity.

Future investigations should also evaluate the pharmacokinetic properties, bioavailability, metabolism, and long‐term safety of afzelin to better establish its in vivo therapeutic potential and clinical translational value. Moreover, exploring possible synergistic interactions between afzelin and other phytoconstituents present in *P. dulce* may provide a more comprehensive understanding of the pharmacological activity of the plant extracts and facilitate the development of effective phytopharmaceuticals.

## Author Contributions

The study was planned and overseen by Md. Ekramul Islam. Masuma Akter conducted the experiment and drafted the manuscript. Mst. Shahnaj Parvin reviewed the draft.

## Funding

No external funding was received for this study.

## Disclosure

The final draft was read and approved by all authors.

## Conflicts of Interest

The authors declare no conflicts of interest.

## Supporting Information

Additional supporting information can be found online in the Supporting Information section.

## Supporting information


**Supporting Information** Figure S1: Raw gel pic indicting the samples from L1 to L7, and boxed lanes are unrelated to the present study, and were not included in the analysis these lanes have not been removed, cropped, or rearranged from their original position on the gel. Table S1: Quantitative analysis of gel band intensities (%) for each band obtained from the gel pic.

## Data Availability

Data are available from the corresponding author upon reasonable request.
