# Supplementary material for: Integrated Analysis of Pithecellobium dulce Leaf Fractions for Ex Vivo Antioxidant, DNA Damage Protective, and Afzelin‐Mediated Anti‐Inflammatory Potentials via In Vivo and In Silico Approaches
Source: Oxid Med Cell Longev. 2026 Oct 19;2026:5529212. doi: 10.1155/omcl/5529212 (PMC13680805; doi:10.1155/omcl/5529212)
Supplement: Supplementary file 1 [file OMCL-2026-5529212-s001.docx]

**L7 L6 L5 L4**

**L3 L2 L1**

**Supplementary Figure S1:** Raw gel pic indicting the samples from L1 to L7, and Boxed lanes are unrelated to the present study, and were not included in the analysis these lanes have not been removed, cropped, or rearranged from their original position on the gel.

**Supplementary Table S1:** Quantitative analysis of gel band intensities (%) for each band obtained from the gel pic

| **Samples** | **Lane** | **Band** | **Band Intensity (%)** |
| --- | --- | --- | --- |
| **Native DNA** | **Lane1** | **Band 1** | **5.113677705** |
|  |  | **Band 2** | **13.5525133** |
|  |  | **Band 3** | **81.33380899** |
| **Control (AAPH Damaged)** | **Lane2** | **Band 2** | **100** |
| **EAF (100 ug/ml)** | **Lane3** | **Band 1** | **1.024002325** |
|  |  | **Band 2** | **10.7151803** |
|  |  | **Band 3** | **88.26081737** |
| **EAF (50 ug/ml)** | **Lane4** | **Band 2** | **3.845834952** |
|  |  | **Band 3** | **96.15416505** |
| **Ascorbic acid (100 ug/ml)** | **Lane5** | **Band 1** | **0.332303018** |
|  |  | **Band 2** | **47.4581633** |
|  |  | **Band 3** | **52.20953368** |
| **CHF (200 ug/ml)** | **Lane6** | **Band 2** | **9.881933332** |
|  |  | **Band 3** | **90.11806667** |
|  | **Lane7** | **Band 2** | **9.16943774** |
|  |  | **Band 3** | **90.83056226** |
